# An equal opportunity for everyone?! Supporting international students of medicine at the Ludwig-Maximilians-University in Munich

**DOI:** 10.3205/zma001201

**Published:** 2018-11-30

**Authors:** Holger Lenz, Wolfgang G. Paik, Fabian Jacobs

**Affiliations:** 1 LMU Munich, University Hospital, Institute for Medical Education, Munich, Germany; 2 LMU Munich, Munich, Germany

**Keywords:** Medical Education, International Students, Communication Skills, Exams

## Abstract

**Objective: **The following article presents OFIF, a project that has been running at the medical faculty of the Ludwig-Maximilians-University in Munich (LMU) since 2013. International students of medicine often lag behind their fellow domestic students in terms of language ability and communication skills. To compensate, OFIF offers communication skills courses with an emphasis on exam preparation. Below, we will discuss the project’s success, challenges and future opportunities.

**Methods:** In their daily academic routine, communication presents one of the greatest challenges for international students of medicine. With the aid of case scenarios and activities from clinical practice, the project courses teach strategies for effective communication. The methodical concept of the OFIF training is based on the six levels of learning of the revised edition of Bloom’s taxonomy.

**Results: **Since 2013, more than 40 trainings and classes for international medical students have been offered. In the winter term of 2017/18, 49 students representing all clinical semesters participated in the OFIF activities. An evaluation of the training classes consistently yielded positive results (95 % of the items were given a score between 9 and 10, on a scale from 1 to 10.)

**Conclusions: **Both the amount of interest (as expressed by the number of actively participating students) and the high percentage of positive evaluations consistently demonstrate that the didactical concept underlying OFIF is useful and could serve as a best practice example for similar projects at other institutions. On the other hand, the 49 participants from all clinical semesters represented but 10 % of the total population of international students in the winter term of 2017/18. Further research examining the concrete effects of OFIF on the academic success of international students at the LMU is therefore desirable.

## Zusammenfassung

**Zielsetzung:** Der vorliegende Artikel stellt das seit 2013 an der Medizinischen Fakultät der Ludwig-Maximilians-Universität existierende Projekt OFIF (Orientierungs- und Fachkommunikationskurse mit Interkulturellem Fokus) vor. Da internationale Studierende der Humanmedizin im Vergleich mit heimischen Kommilitonen oft Defizite in den Bereichen Kommunikation und Sprache vorweisen, bietet OFIF Kommunikationstrainings mit prüfungsrelevanten Schwerpunkten an. Im Folgenden werden Erfolge, Herausforderungen und Chancen des Projekts erläutert.

**Methodik: **Kommunikation ist eine der größten Hürden im Studien- und Prüfungsalltag internationaler Medizinstudierender. Daher vermitteln die Trainings des Projekts Kommunikationsstrategien anhand von Fallbeispielen und klinisch-praktischen Übungen. Das methodische Konzept der Trainings basiert dabei auf den sechs Lernebenen des kognitiven Modells der revidierten Taxonomie nach Bloom.

**Ergebnisse: **Seit 2013 wurden über 40 Trainings und Kurse für internationale Medizinstudierende angeboten. Im Wintersemester 2017/18 nahmen 49 Studierende aus allen klinischen Semestern an OFIF-Kursen teil. Die Bewertung der Kurse durch die Teilnehmer war stets positiv (95% der Bewertungsitems wurden auf einer Skala von 1-10 mit 9 oder 10 bewertet).

**Schlussfolgerung: **Die Tatsache, dass Studierende das Angebot von OFIF von Semester zu Semester immer wieder wahrnehmen und positiv bewerten zeigt, dass es sich hier um ein sinnvolles Konzept für die Unterstützung internationaler Studierender handelt, das als best practice Beispiel dienen kann. Gleichzeitig repräsentieren die 49 Teilnehmer im Wintersemester 2017/18 lediglich 10% der im klinischen Semester eingeschriebenen Studierenden aus dem Ausland. Weitere Forschungsarbeiten, die sich mit der Frage nach der tatsächlichen Auswirkung der Kurse auf die Prüfungs- und Studienleistungen internationaler Studierender beziehen, sind geplant.

## 1. Introduction

A degree in medicine from an institute of higher education in Germany remains a popular choice among high school graduates and university students today. This has shown to be true for international students coming to Germany as well as for their fellow domestic students. According to data published by the Federal Statistical Office, the percentage of international medical students in Germany increased by nearly 20% between 2012 and 2016 [1]. Although Chenot et al. pointed out as early as 2007 that a significant void exists within the research area of “foreign medical students in Germany”, no systematic examination of this group has been undertaken to this day. Subsequently, a standardized, problem-oriented repertoire of support structures for this group has not been established at institutions of higher education in Germany [2]. Furthermore, complaints about perceived language barriers, a lack of orientation within the academic system, financial difficulties, and apartment-hunting abound among students as well as lecturers [3]. According to a study conducted by the Institute for Medical and Pharmaceutical Examination Questions (IMPP) in 2013, 15-60% of all international students (with variations between the respective countries of origin) fail to pass the first state examination at first attempt, compared to just 10% of their German co-students [4]. International students also perform worse in the second state examination, and, on average, finish medical school three semesters later. Both the higher exam failure rates and the lower examination grades were significant. 

Support structures, if available, are often based on the perceptions of academic administrators and do not meet the real needs of international students. At the same time, academic institutions face the challenge of presenting solutions to a broad spectrum of different problems and needs. Therefore, support usually addresses one or two of the above mentioned issues [5]. At the Ludwig-Maximilians-University of Munich (LMU), project OFIF aims to address these issues by offering tailored trainings for international students. Improving communication at school and in the professional clinical environment turned out to be a major need for the group of international students. Thus, teaching verbal and non-verbal strategies that international students can apply both during exams and in actual conversations with patients has become a major focus of the project.

## 2. Project Description

The following is a description of the OFIF project from its inception to its current form.

### 2.1. Needs Analysis

Based on the above mentioned lack of research about the state of international medical students in Germany, OFIF was founded in 2013 in order to better understand the needs of this population at the medical faculty of the LMU. A comprehensive needs analysis including guided interviews with lecturers (N=11) and international students (N=9) as well as an online survey for both groups (Lecturers: N=67, Students: N=100) was conducted at the medical faculty. 

The guided interviews yielded the following results:

Lecturers reported that international students experience difficulties with:

- Communication with patients, especially when required to switch between every-day speech (vernacular) and medical-technical vocabulary
- Issues with verbal expression and comprehension of questions and/or instructions during oral and practical examinations
- From the lecturers perspective, many of the international students also appeared to be socially isolated, participating in study groups less often than their fellow German students

International students reported experiencing difficulties with:

- Accurately conveying their answers during oral examinations
- Understanding examination questions on written Multiple Choice exam papers
- Social integration into study groups and friendships with German students in general
- Especially during the first years at the university, many international students also either wished or would have wished for a tutor-/study buddy-system that facilitates their orientation within the school administrative system and the acquisition of proper studying techniques

#### 2.2. Extent of Classes Offered

Based upon the results of the needs analysis, OFIF decided to introduce a tripartite series of trainings (each part consisting of 2 to 4 individual lessons) including a total of five practice- and communication-focused trainings (cp. Table 1 (Tab. 1)). Registration for and participation in each training of the series was voluntary and not part of the standard medical school curriculum. Due to great demand, two additional trainings focusing on multiple choice exams and oral exams respectively were added to the series during the year of the project’s inception. All trainings were initially open for international medical students of all semesters.

Goal of the trainings was the transfer of knowledge and the exchange of experience between students of different semesters. International students in their pre-clinical years were afforded the opportunity to benefit from the experience of senior students. More advanced students received an opportunity to function as study buddies, tutors or mentors, applying their own experience to help other students adapt to the foreign country and school more easily.

##### 2.2.1 Methodical Implementation

Methodically, the trainings were based on the concept of action-oriented learning within simulated environments [6]. Two trainings dealing with the topic of “oral examinations” were offered to the international students. This examination type presents a great challenge for many international students, as they are confronted with increased nervousness and difficulty in expressing themselves with ease. Furthermore, past research has shown that students with different cultural backgrounds can sometimes be used to different styles of studying, too, impeding their academic success in new cultural environments [7]. In the classes offered, international students were taught study strategies commonly used by successful students at German medical schools, as well as presentation skills and verbal/non-verbal behavior guidelines for oral and practical examinations. Theoretical knowledge was made practical by offering students the opportunity to participate in simulated oral examinations, where they were able to safely practice their newly gained skills and knowledge, receiving feedback from peers and tutors.

Furthermore, two trainings on the topic of “Multiple Choice Questions” were offered. The complexity of the language and grammar commonly used in this question type presents a challenge for many international students. By teaching the students study strategies for this type of exam, OFIF aimed to facilitate the international students’ adaptation to this particular type of exam. 

Finally, after successfully mastering acquisition of professional medical terminology, many students find it a challenge to communicate successfully with patients. In the OFIF training, international students therefore focused on appreciating the differences between common, ever-day speech and medical terminology, practicing both in simulated doctor-patient-conversations [8].

##### 2.2.2 Evaluation

At the end of each OFIF training, participants were asked to fill out a feedback survey in order to enable the project team to continuously learn about the actual (and changing) needs of the international student community and to adapt the course offerings accordingly. In the winter term of 2013/14, a total of 65 participants (52 full-time and 13 Erasmus-students) from 27 countries participated in the five trainings. 38 of them were students in their pre-clinical semesters. 27 had entered the clinical part of their studies. On average, the students evaluated the trainings with 1.4 on a scale of 1 (very good) to 5 (very bad).

#### 2.3 The current project

Starting with the winter term of 2015/16, the homogeneous approach of coaching students of all semesters within the same classes was abandoned in favor of a split approach respecting the different technical and communicative skill levels of the participants (cp. Table 1 (Tab. 1)). For students in their pre-clinical semesters, a separate, newly founded project has been offering classes since, while OFIF has kept assessing and meeting the needs of international students in the clinical part of their studies.

Since oral and practical examination formats dominate the clinical part of medical school (e. g., OSCEs at the LMU), OFIF discontinued the offer of trainings aimed at preparing students for written examinations (multiple choice examinations). Instead, trainings aimed at preparing students for oral and practical examinations were extended in their content, and specifically re-structured to prepare students for concrete situations they can expect to encounter during their academic and professional education. During the winter term of 2017/18, the following trainings were offered:

1. Communication skills training *Taking A Patient’s History* (2 hours)
2. Communication skills training *The Physical Examination* (1.5 hours)
3. Communication skills training *Patient Education Before A Surgery* (1.5 hours)
4. Communication skills training *A Difficult Patient* (1.5 hours)
5. Communication skills training *The Digital-Rectal Examination* (1.5 hours)

The trainings were conducted on a Tuesday evening, every week for five weeks in a row, following the order in which they are listed above. The series was offered once per semester. Participants were required to be enrolled at the medical school of the LMU, and to speak German as a foreign language. The trainings were promoted during introductory lectures at the beginning of the semester, advertised using flyers, social networks (Facebook), as well as the medical faculty’s email newsletter. Registration was to be completed using an online form, as the maximum number of participants was limited to 21 students per training session for didactical reasons. Each training, with the exception of *Taking A Patient’s History*, lasted for 90 minutes. The following section explains the general structure of the OFIF trainings as well as the underlying didactical basis in more detail.

##### 2.3.1 Methods

The main goal of each OFIF training is the creation of a controlled learning environment for participating students, in which the latter are afforded an opportunity to gain awareness of, reproduce, and critically reflect aspects of their own communication skills in an explicit way, which, in the clinical context, would otherwise only have been taught implicitly. Didactically and structurally, each OFIF training is based on the principles introduced in Bloom’s revised taxonomy [9]. A great strength of Bloom’s taxonomy is that it establishes a clear relationship between learning activity and learning success [10]. Bloom’s six levels of learning and their respective practical realizations within the typical OFIF training session shall be discussed below (cp. Table 2 (Tab. 2)). 

In contrast with Bloom’s original taxonomy, the revised version is marked by the deliberate use of verbs and gerunds for the description of each of the six levels of learning. The use of these so-called action words stresses the significance of the cognitive processes enabling students to encounter and process the knowledge they seek to acquire [11]. The use of the action words also leads to a better description of the nature of the learning processes, whose successful completion entails the successful acquisition of a set of freely deployable skills rather than the mere acquisition of a fixed amount of crystallized and static knowledge [9], [11]. The six levels as described in the revised taxonomy are:* Remember, Understand, Analyze, Apply, Create, Evaluate.*

##### 2.3.2 The Structure of An OFIF Training And Its Methodical Fundament

During the first five minutes of each OFIF training session, participants introduce themselves to the group. A short inquiry as to prior experiences with the theme of the training offers an opportunity to discuss the practical relevance of that theme. Introductions are followed by a formal presentation of the session’s theme by the instructor. This includes a 20-minute PowerPoint presentation, in which necessary medical technical knowledge is reviewed and taught. During this step, active engagement of the students is key; it is realized by actively encouraging the students to share relevant (previous) knowledge with the group (Remember). A concrete example would be to ask a student to remember and explain the concept of the communicative technique of *Echoing* during this first phase of the class.

After the instructor’s presentation, the acquired knowledge is reviewed and re-organized. 

In the example of *Echoing,* students could be asked to compare the technique of *Echoing* with the (newly learned) communicative technique of *Paraphrasing*. Cognitively, this practice is aimed at producing a clearer separation between the two terms, which, in turn, results in a clearer understanding (*Understand*) of them.

In the next stage, students form small working groups of two or three and complete a fill-in-the-blanks exercise. This activates their newly acquired knowledge (e.g. the *Echoing* technique). From a didactical perspective, the fill-in-the-blanks exercise sets the tightly controlled perimeter in which learners practice to retrace and apply isolated parts of their knowledge (*Apply*). Within this step, the maximum degree of didactically planned isolation of singular conversation fragments is reached – the only flexible, or moving parts in the setting being the skills applied by the learners themselves.

Afterwards, a ten minute long sample conversation between a doctor and a patient (performed by the instructors) re-introduces complexity and brings the learner back to the reality of clinical practice. The goal of this performance is to let the students experience the theoretically acquired knowledge in a realistic practical setting. Using their theoretical knowledge, students are then asked to dissect the scenario into categories of information and to analyze it for those elements of communication that have previously been introduced theoretically. A concrete example would be to let students examine the scenario with the goal of finding all instances where the communicative technique of* Echoing* has been applied (*Analyze*).

During the final phase of the training session, students are directly confronted with a scenario taken from clinical practice. Students are asked to form working groups of three and are given a session-specific scenario description accompanied by a checklist that fits the scenario. Each group proceeds to play out the scenario three times, with every student taking on the role of doctor, patient, and observer exactly once. The observer’s role in each round is to observe and assess the communicative behavior of the student playing the doctor by means of the checklist. For example, the observer checks whether the doctor succeeded in making correct use of the communicative technique of* Echoing*. The difference between prior exercises and this one is that this exercise asks the students to actively create for themselves the context in which they can embed their newly acquired skills. It serves the active integration of the newly acquired skills into the students’ behavioral repertoire, and it fortifies their skills by letting them experience an almost identical situation three times. After concluding this exercise, the entire class regroups and discusses perceived challenges and overall performance of participants (*create and evaluate*). Table 2 (Tab. 2) offers a tabular overview of the described structure.

#### 2.4 Organization of the Project

Since 2015, OFIF has been cooperating with the Study-Buddy program at the medical faculty of the LMU. The Study-Buddy program, aimed at Erasmus-students spending one or more semesters at the medical faculty of the LMU, pairs each international Erasmus-student with a domestic student who takes on the role of a tutor or “buddy”. Erasmus students are welcome to join the non-mandatory OFIF trainings, creating an opportunity for full-time international students and Erasmus-students to share experiences and knowledge with each other. Under the didactical supervision of OFIF instructors, two of the OFIF training sessions,* Taking A Patient’s History *and *The Physical Examination* were led by members of the Study-Buddy program in the winter term of 2017/18. Additional cooperation with other institutions and programs appears desirable at this point in time. Not only may such cooperation benefit the international students by widening the spectrum of support offers available to them, but it would increase the visibility of the OFIF project, making it easier for the project to reach larger portions of the target audience.

##### 2.4.1 Project team

Since 2013, the OFIF project team includes one Research Associate with a background in Educational Sciences (part-time, 50 %) and two student Research Assistants from the medical faculty (8 hours per week each). All trainings sessions were developed and implemented by the Research Associate and the two Student Assistants. Physicians and surgeons at the University Hospital of the LMU were consulted as needed.

Since its inception, the project has been funded through grants from the *Committee For The Distribution Of Funds From Tuition Fees* of the medical faculty at the LMU. With a view to the university-funded project *Lehre@LMU* (*Teaching@LMU*), the project team has applied for further grants for 2018, which were granted. Commencing in January 2018, three student Research Assistants will be working on the project.

## 3. Results

In the winter term of 2017/18, 55 students attended an introductory event held in the week immediately preceding the first training session. The programs main goals and the concept of the training sessions were introduced during this event. The 55 students that were in attendance that day represent 10 % of the target audience of all international medical students in the clinical section of medical studies at the LMU (N=543). The training sessions themselves received 91 registrations and were attended by 49 students. Students were free to attend all or just a number of the sessions offered in the semester series. The training sessions *Taking A Patient’s History* gathered the greatest attendance (16), followed by* Patient Education Before A Surgery* (12 participants), as well as *The Physical Examination and A Difficult Patient* (8 participants each). The training session *Digital-rectal Examination* was attended by 5 students. The country most often represented by the participants was Turkey (9), followed by Hungary (6), Israel, Belgium, Greece (4), and Italy, Russia, Slovenia, Cyprus (3). Bulgaria, Finland, France, Cameroon, Libya, Poland, Serbia, Spain, and Vietnam were represented by one student each. One German student also participated in the trainings.

Since the training sessions *Taking A Patient’s History* and *The Physical Examination* were not conducted by the OFIF team, but by the Study Buddy team, no evaluations for these two sessions exist. Overall, both anecdotal and statistical evidence (cp. Table 3 (Tab. 3)) show that participants evaluate the OFIF trainings positively. It is especially noteworthy that 90% completely agree that they would recommend OFIF training sessions to others. 

## 4. Discussion

Throughout its five-year history, project OFIF has received very good evaluations from the international student community at the medical faculty of the LMU. It has established itself as an integral part of the service structure at the faculty. The above mentioned attendance figures show, however, that there is a large correlation between the date of a training session and student participation: the earlier the training in the semester, the more students attend. For this reason, a tentative cooperation with the Intercultural Counseling Service (ICS) at the LMU had to be discontinued. 

At this moment, it is not clear what contributes to the difference in attendance figures throughout the semester. It is noteworthy that not every students who registers for a training sessions actually attends the session. Out of 94 registrations, only 49 led to actual attendance. It is not clear why this was the case. The growing pressure to prepare for exams, or even a growing indifference towards one’s own language abilities might have played a role. Anecdotic reports from students suggest that some international students had heard of project OFIF, had made plans to attend a training session, but still ended up not attending because of scheduling difficulties or personal reasons. A study involving a focus group has been planned to further examine the reasons for this phenomenon.

## 5. Conclusion

In May 2018, project OFIF will have celebrate its fifth anniversary. This year, therefore, offers the project team an opportune occasion to conduct a comprehensive project analysis and to examine whether the project has succeeded in contributing to a reduction of some of the many hurdles for international students studying at the medical faculty that were brought to light by the needs analysis of 2013. At the time of this report, it is not yet clear how and in what way the project has contributed to the improvement of their situation. Based on the numbers presented, it is very clear that, despite the projects’ efforts to address the real needs of the international student community and to promote the project by attending introductory lectures, participating in social media, and using email newsletters, much work remains to be done. A total of 490 full-time international students were enrolled in the medical program of the LMU during the summer term of 2017 – 49 students participated in OFIF trainings in the winter term of 2017/18. Finding a way to reach and involve a greater percentage of the international student body without making the project’s classes obligatory is going to be a crucial task for the future.

Experience has shown that it is difficult, impossible perhaps, to find a comprehensive solution that addresses all challenges and problems that were mentioned in the introduction of this article. Uncertainty regarding the financial backing of the project makes it difficult to plan and realize more diverse, more comprehensive approaches and ideas. Only if the issues surrounding international students continue to gain visibility among administrators, perhaps in the form of a permanent, centralized support center at the medical faculty, will the international student body profit fully from our efforts.

## Acknowledgements

Project OFIF acknowledges the funding it has received from the tuition fees fund of the medical school at the LMU.

## Competing interests

The authors declare that they have no competing interests. 

## Figures and Tables

**Table 1:**
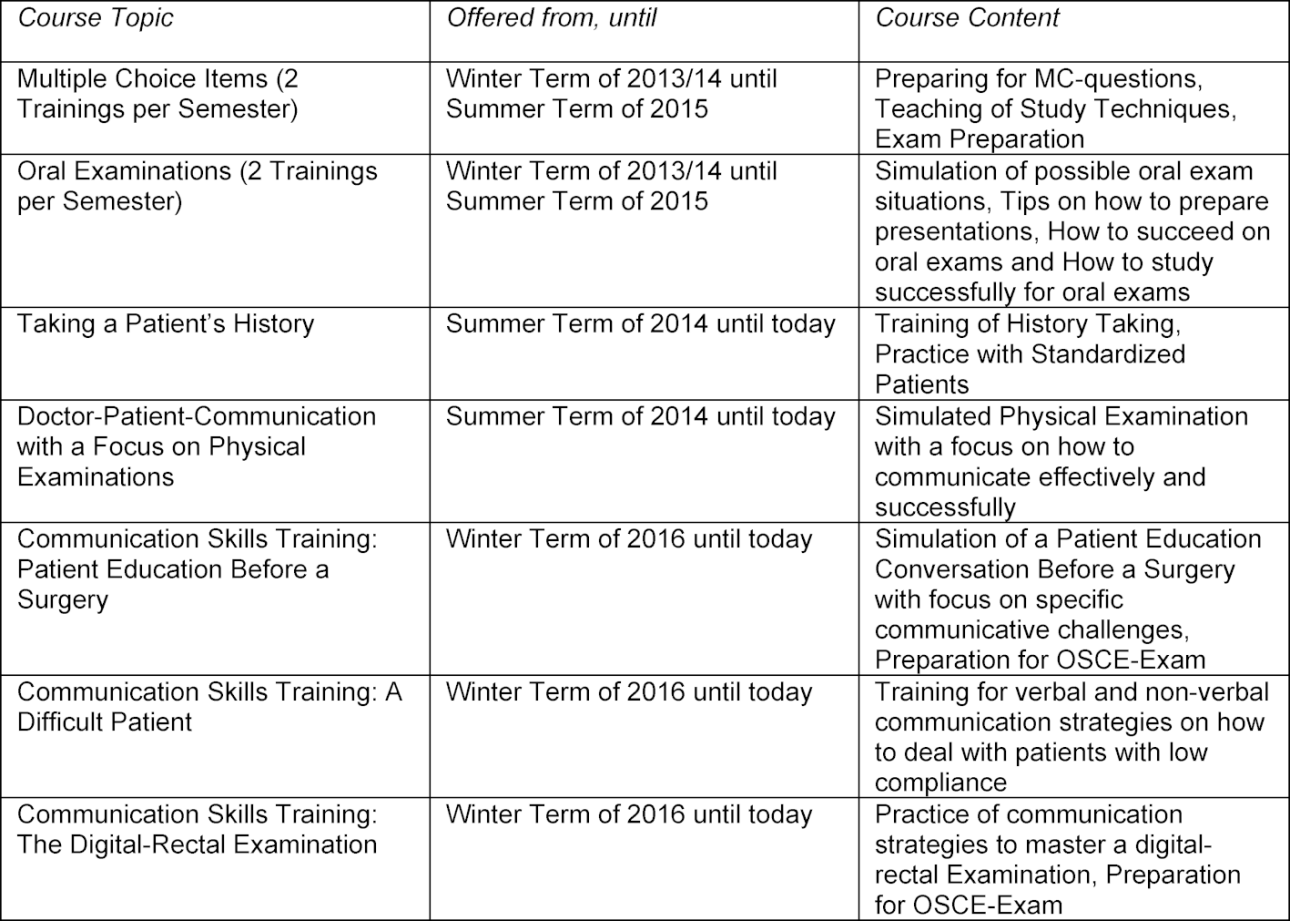
List of OFIF course offerings from Oct. 2013 until today

**Table 2:**
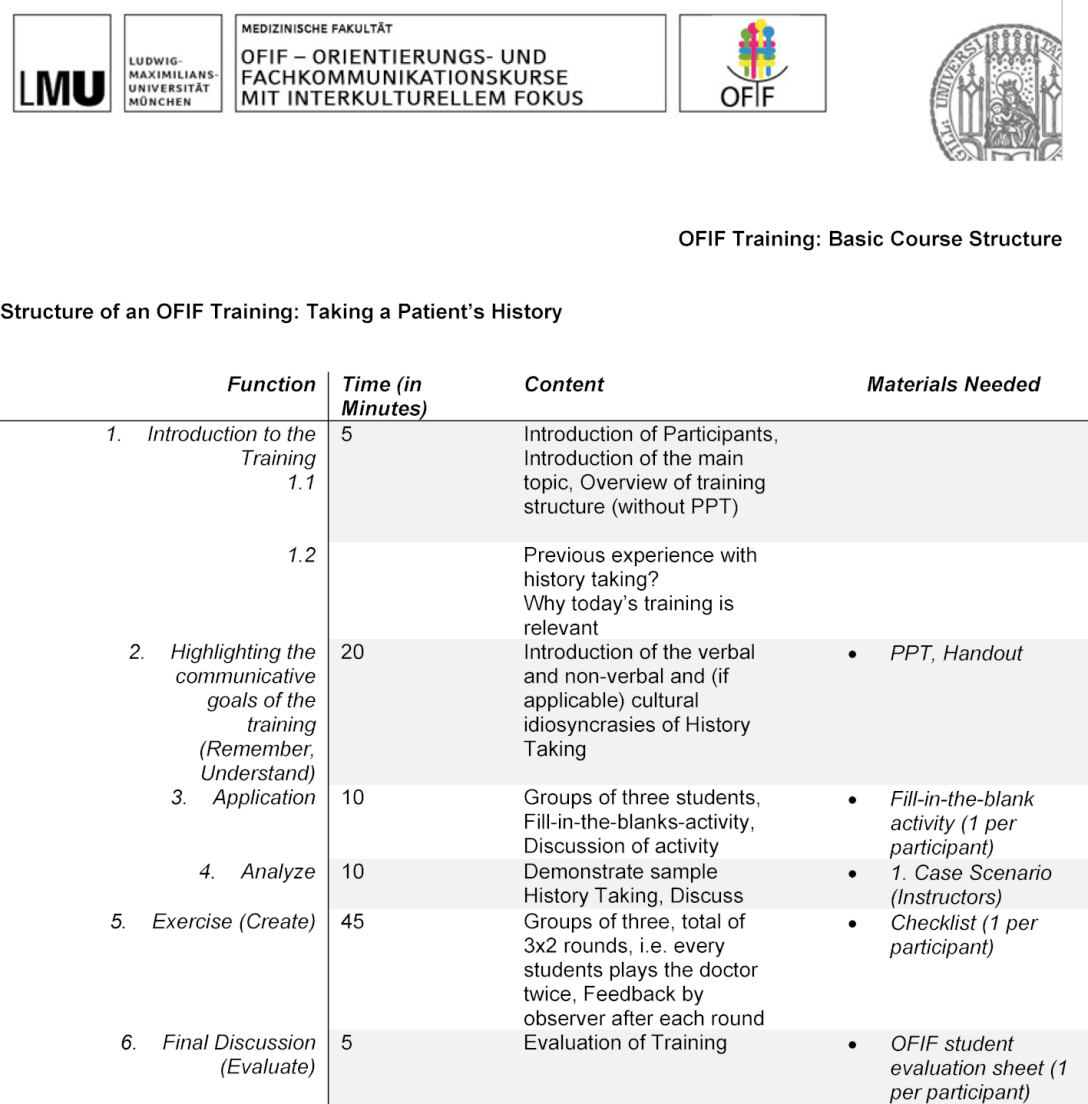
Basic structure of an OFIF communication training course

**Table 3:**
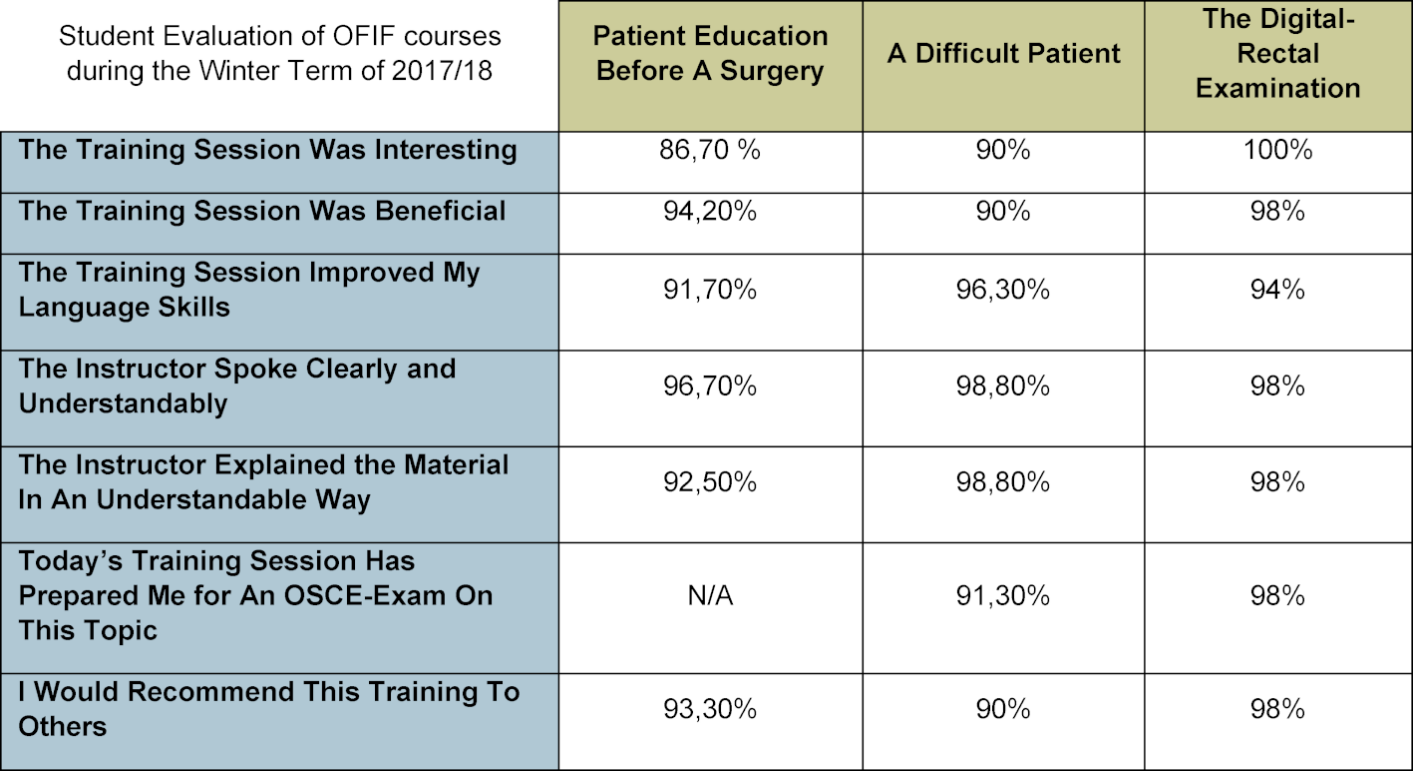
Student evaluation results for the 2017/18 OFIF communication trainings program. The item “Today's training session has prepared me for an OSCE-Exam on this topic” was added after the training “Patient Education Before A Surgery”
